# Process evaluation of integrated early child development care at private clinics in poor urban Pakistan: a mixed methods study

**DOI:** 10.3399/bjgpopen17X101073

**Published:** 2017-10-04

**Authors:** Muhammad Amir Khan, Syeda Somyyah Owais, Sehrish Ishaq, John Walley, Haroon Jehangir Khan, Claire Blacklock, Muhammad Ahmar Khan, Muhammad Waqar Azeem

**Affiliations:** 1 Chief Coordinating Professional, Association for Social Development, Islamabad, Pakistan; 2 Project Manager, Association for Social Development, Islamabad, Pakistan; 3 Project Coordinator, Association for Social Development, Islamabad, Pakistan; 4 Professor of Internal Public Health, Nuffield Centre for International Health and Development, Leeds Institute of Health Sciences, University of Leeds, Leeds, UK; 5 Provincial Coordinator (Non-Communicable Diseases & Mental Health), Directorate General of Health Services, Lahore, Pakistan; 6 Lecturer in International Public Health, Nuffield Centre for International Health and Development, Leeds Institute for Health Sciences, University of Leeds, Leeds, UK; 7 Research Associate, Association for Social Development, Islamabad, Pakistan; 8 Chair of the Department of Psychiatry, Sidra Medical and Research Center/Weill Cornell Medical College, Cornell University, Doha, Qatar

**Keywords:** primary health care, private facilities, public-private partnerships, Pakistan, child development

## Abstract

**Background:**

In poor urban Pakistan, private GP clinics lack adequate services to promote early child development (ECD) care. A clinic-based contextualised ECD intervention was developed for quarterly tool-assisted counselling of mothers.

**Aim:**

To explore the experience and implementation of ECD intervention by the private care providers and clients, for further adaptation for scaling of quality ECD care, at primary level private healthcare facilities in Pakistan.

**Design & setting:**

A mixed methods approach using quantitative records review and qualitative interviews at poor urban clinics in Rawalpindi and Lahore, Pakistan.

**Method:**

Quantitative data from study-specific records were reviewed for 1242 mother–child pairs registered in the intervention. A total of 18 semi-structured interviews with clinic staff, mothers, and research staff were conducted at four clinics. The interviews were audiorecorded and transcribed verbatim.

**Results:**

District Health Office (DHO) support allowed transparent and effective selection and training of clinic providers. Public endorsement of ECD care at private clinics and the addition of community advocates promoted ECD care uptake. Clinic settings were found feasible for clinic assistants, and acceptable to mothers, for counselling sessions. Mothers found ECD counselling methods more engaging compared to the usual care provided.

**Conclusion:**

In poor urban settings where public health care is scarce, minimal programme investment on staff training and provision of minor equipment can engage private clinics effectively in delivering ECD care.

## How this fits

Most ECD promotion in Pakistan has been focused through community-based interventions. The implemented trial investigated clinic-based, integrated ECD delivery at private GP clinics. Process evaluation of the intervention care components will inform the mechanism for scaling up of ECD programmes under public–private partnership.

## Background

ECD delay is a grossly neglected public health problem. During the initial 36 months of life, the brain is highly responsive to the surrounding environment which requires appropriate nourishment and stimulation to reach full developmental potential.^1^ Lack of stimulation, attachment issues, recurrent infections, poverty, and inability of the parents to provide appropriate care can impede a child’s subsequent emotional, social, communication, and cognitive development contributing significantly to delayed achievement of early childhood developmental milestones.^2–6^

In poor urban areas of Pakistan, private GP clinics are the usual providers of primary care, due to the lack of public services. The usual maternal and child health services provided by these facilities do not adequately support and promote ECD. Consequently, integrated educational counselling for mothers is lacking, mainly due to unavailability of contextualised clinical tools, and inadequate skills and engagement of clinic staff. However, district-led public–private partnership initiatives do exist for delivering tuberculosis, malaria, diabetes, and hypertension care in Pakistan.^7–9^

A contextualised ECD intervention was developed through a programme-led technical working group. The intervention and trial design are fully described in the published trial protocol.^10^ In brief, the core intervention was a structured clinic-based, quarterly tool-assisted counselling session for mother–child pairs (Box 1). Clinic assistants in the intervention arm offered each mother a structured 10-minute counselling session when her child was <6 weeks old, and again at 3 months, 6 months, and 9 months of age. Each counselling session covered age-appropriate activities to promote early child nutrition and stimulation, and maternal mental health. Public–private partnership and community advocate activities were undertaken in both arms of the trial.

**Box 1.** Early child development care in private GP clinics (adapted from the published protocol).^10^

	Intervention clinics
Mother–child care^a^	Standard counselling session using pictorial flipbook: childhood nutrition development maternal mental health Assessment and treatment (including referral to specialist) of childhood nutrition, development, or maternal depression. Follow-up of mother–child pairs in clinic at 3, 6 and 9 months (including SMS or telephone reminder, if required)
Training of private doctors and clinic assistants	Clinic assistants training: Conducting a standardised counselling session using the flipbook Measurement and recording child length and weight Private GPs training: Clinical management of children with malnutrition and developmental delays in the private clinic setting. How to assess the mother–child pair for a specialist referral, when required, to the appropriate public tertiary care facility.

^a^Control clinics continued with usual routine care.

The structured counselling intervention was aimed at enhancing maternal skills, by performing a set of specific activities with the child at home to achieve age-appropriate developmental milestones to reduce the incidence of developmental delays in early childhood. It also promoted childhood nutrition in the home, and maternal mental health. It was evaluated using a randomised controlled study design, in 16 intervention clinics clusters and 16 control clinics, in poor urban settlements of districts, Lahore and Rawalpindi, Pakistan. A total of 2327 mother–child pairs were registered in the trial. The intervention was found effective in achieving reduction in the development delays (at 12 months of child age) among registered children (results are currently being submitted for publication).

During the process of developing the intervention, certain assumptions were made about the circumstances and behaviour of health facility staff and mothers, which may or may not have been experienced in practice (Box 2). Evaluating the experiences of trial implementation and fidelity identifies contextual factors and causal mechanisms for variations and further adaptations.^11^ A process evaluation was therefore designed, and conducted alongside the main trial, to: a) allow better understanding of on-the-ground intervention delivery experiences (such as, feasibility and acceptability for both facility staff and mothers); b) explore any deviations in care practices from trial protocol by clinic staff (such as, design or implementation failure); and c) inform future scaling of the intervention.

**Box 2.** Logic model summary (adapted from the published protocol).^10^

	Inputs	Processes and actions	Intended		
			Changes	Outputs	Outcomes
Intervention	Contextualised counselling tool Training of private GPs and clinic assistants – technical and practical training	Quarterly counselling for mothers (child development, child nutrition, maternal mental health) Assessment and treatment of child developmental delays (may include referral)	Clinic staff knowledge, skills, and resources	ECD care delivered, as per protocol, at the clinics ECD care uptake by mothers	Reduced child developmental delays at 12 months of age
Both intervention and control arms	District endorsement Community advocates Child Growth Cards Infant weighing scale and infantometer	Recruitment of mother–﻿child pairs referred by community advocates		Adequate participant recruitment and recording data	

ECD = early child development.

The main aims of the process evaluation were therefore:

- to explore how intended intervention components were implemented and experienced by the private care providers and clients, at poor urban private GP clinics; and
- to identify how intervention components could be further adapted for scaling of quality ECD care, at primary level private healthcare facilities in Pakistan.

(The process evaluation of nutrition and maternal mental health components has not been reported in this article and will be published separately).

## Method

### Study design

A mixed methods design was used to explore the implementation of the ECD intervention, combining both quantitative records review, and qualitative interviews.^12^ Quantitative records of mother–﻿child pairs registered in the intervention arm, were reviewed mainly to learn about the coverage and delivery of ECD care tasks including child physical measurements, follow-up, and record keeping. Qualitative interviews were used for in-depth enquiry into the fidelity of the intervention components, including the role of individual and contextual factors. In addition, certain aspects of the trial which were implemented in both arms (for example, public–private partnership and community advocates) were also explored in interviews.

### Participants

Quantitative data were reviewed for all 1242 mother–child pairs registered at the 16 intervention clinics. Participating clinics fulfilled trial eligibility criteria, which included years of experience and current patient load.

The intervention clinics in both districts were divided into high-performing or low-performing groups, based on client registration volume, client adherence to follow-up visits, and clinic performance indicators (for example, record keeping) during the follow-up visits. In each of the two districts, one high-performing and one low-performing clinic was randomly selected for qualitative data collection by drawing from a hat at random. At each of the four selected clinics, two staff members (a doctor and a clinic assistant) and two mothers (every first and last mother coming for outcome measurement) were interviewed. Two members of the field implementation team (that is, one in each district), with hands-on experience of monitoring ECD care at the clinics, were also interviewed. This led to a total of 18 semi-structured interviews with clinic staff, mothers, and research staff. The number of individuals interviewed, in various responder categories, reflected an acceptable balance between scientific and feasibility considerations.

### Data collection

#### Quantitative data

Study-specific child growth cards, maintained by staff at each participating clinic (from May 2015 to September 2016) were used as the source of quantitative data for selected indicators. At the completion of trial, the paper records (cards) maintained for each child were collected from the respective clinic.

#### Qualitative data

Semi-structured interviews were conducted at selected clinics, by two trained female researchers. A standard topic guide was used in all interviews. Each interview was conducted at the time of trial outcome measurement (at 12 months), after verbal consent, and lasted no more than 30 minutes. The interviews, conducted in national/local language (Urdu), were audiorecorded. Some responders were also subsequently called by telephone, when needed, to clarify or elaborate on their initial responses.

The collected data were further supplemented by review of project implementation and monitoring records.

### Data analysis

#### Quantitative

Quantitative data were entered directly into SPSS (version 17) and frequencies were analysed for each selected indicator.

#### Qualitative

A Framework Approach to qualitative analysis was used.^13,14^ The same two researchers conducted and transcribed all interviews. Transcripts were anonymised, and identifiable participant information was stored separately and securely. After repeated reading of the transcribed data, the researchers were familiarised with the data and identified iterative codes. These emergent codes were added to a pre-defined coding framework considerations (organisational, case management, continuity of care (Box 3), and the modified framework was applied to the rest of the data. Themes were analysed and interpreted through back-checking transcripts for consistency; triangulation of data collected from different participant groups (for example, providers and clients) and ongoing discussion within the research team on the relevance to programmatic scaling.

**Box 3.** ECD task categories, indicators and data collection methods.

Task categories	Process indicators and data collection methods	
	Quantitative	Qualitative
**Organisational**		
Provider engagement and public–private partnership	Number of clinics reviewed and short-listed for selection Number of clinic doctors and clinic assistants trained	Types of clinics suitable for ECD care Objectivity and transparency of clinic selection
Community engagement through community advocates	Number of community advocates recruited for ECD care promotion Number/proportion of mother–child pairs referred by community advocates	Types of advocates engaged Enabling of advocates
Other	Attrition of clinics form ECD care intervention Number of monitoring visits	Any subsidy (by private clinics) on ECD care visits
**Case management**		
Care delivery by private clinic assistant (structured counselling)	Number of mothers counseled	Integrating ECD counselling in routine care Use of pictorial tool for mother counselling
Care delivery by private clinic doctor	Number of children identified with development delays	Child development assessment and recording
**Continuity of care**		
Follow-up by private clinic	Number of mother–child pair attending quarterly follow-ups Relationship between adherence and child development	Mothers adherence to follow-up visits Clinic staff action for better follow-up adherence
Referral by private clinic	Children referred for specialist care	Referral arrangements: access and cost Referral experiences of clinic staff and mothers

ECD = early child development.

#### Integration of mixed methods data

Quantitative process evaluation findings were used to inform the interpretation of qualitative results, and vice versa. Integration of quantitative and qualitative findings was done at the analysis and interpretation phase.

## Ethical considerations

Through facilitation of the two respective DHOs, consent of the selected clinics was obtained. At the time of registration, each mother was informed about the trial and consent (written) was obtained including use of their records. At the time of outcome measurement, consent was obtained from each of the eight mothers interviewed for process evaluation exercise. Data management procedures were followed to ensure confidentiality of recorded quantitative and qualitative data sets.

## Results

### Organisational

#### Provider engagement and public–private partnership

Three clinics in each selected cluster (union council) were initially shortlisted based on number of years since established, patient load, set of services offered, and willingness to participate in ECD care activity. One clinic in each union council was then selected on the basis of surveyed indicators, district official inputs and clinic consent to participate. A few initially shortlisted clinics were unwilling to participate because of heavy patient load and shortage of time to commit to the proposed ECD care. The transparent and objective selection of private clinics, with support of the DHO, helped credibility by limiting potential personal influences/preferences:

*'Surveying and scored-based selection of clinics has helped minimising individual biases in the selection process.'* (Field coordinator [FC]1)

Planning the timing of the training session according to the private clinics’ working schedules was important for effective training and to ensure attendance. The providers preferred attending a session during their off-time for no longer than 2 hours. The venue and date for the training event were decided in consultation with the respective district health official and the institution staff. A formal invitation letter from the respective DHO and reminder calls by the project staff helped achieving more than 90% (58 out of 64) attendance of the invitees. The utilisation of trainers from public psychiatry or child development institutions, and the district level training setup (District Health Development Center), also helped in encouraging invitees, maintain quality and build district-level capacity. The trainees found the programme-endorsed training, with its concise content and focus on application of care tools, helpful in their learning:

*'We enjoyed learning new skills; made it simple for us to deliver ECD care in real-life situation.'* (Clinic assistant [CA] 4)

No financial incentive was offered for the clinics to participate in the trial; however, most private doctors showed interest in their clinic getting branded for ECD care by the district, because of the perceived advantage of being a public-recognised service provider:

*'Public branding gives us competitive advantage over other clinics.'* (Clinic doctor [CD] 3)

However, an official government branding was not feasible for an early-stage innovation (that is, the intervention being piloted and evaluated for generating evidence); instead a poster with public-endorsed ECD care information was developed and displayed at each clinic. Due to multiple challenges (including space, legal requirement, and tax implications), almost all clinics displayed the ECD posters inside their premises.

#### Community engagement through community advocates

About one-quarter (570 out of 2327) of total mother–child registration for ECD care at private clinics were found to be advocate-referred (that is, the advocate informed and encouraged the parents to access ECD care). The process of selection of institution-based advocates (for example, grocery shop, drug store, or mosque) was challenging due to lack of existing data on these outlets, and less clear criteria to select one out of the few existing. The selection of position-based community advocates (for example, vaccinator or community worker) was found to be relatively easier (due to existing data) and helpful because of their frequent interactions with mothers and families:

*'Addition of advocates with a healthcare background supplements the working of non-healthcare community advocates.'* (FC 2)

### Case management

#### Care delivery by private clinic assistant

The clinic settings were found workable for clinic assistants and acceptable to mothers, for a counselling session. The choice of clinic seemed to indicate a perceived match between women's expectation (of health care) and the specific clinic setting. Except for a few participants with conservative social backgrounds, most mothers attended the clinics unaccompanied.

Using pictorial counselling tool was found helpful and acceptable. Mothers found this method more engaging as compared to the usual 2-minute verbal interactions at clinics. The pictures helped mothers understand and remember the advice more clearly. Some women reported that the take-home leaflet was a helpful memory aid which helped in sharing the messages with other family members. Some also mentioned using the brochure for child care during the subsequent months, even when they were not able to attend the clinic:

*'Simple pictures helped me perform the actions recommended for my child.'* (Mother 1, Clinic 3)

The clinic assistants found mothers with few years of formal schooling more receptive and engaged during the counselling. However, the staff found it difficult to counsel a mother whose native language was different; in such cases the accompanying person would aid in the counselling process:

'Sometimes *a person accompanying mother helped in overcoming the language barrier.'* (CA 3)

#### Care delivery by private clinic doctor

Doctors were found inclined to focus on carrying out the clinical tasks (for example, child clinical assessment in case of a reported delay) and delegate other care tasks to clinic assistants. This was done to achieve efficiency through delegation of the care load:

*'Shifting the patient education task to clinic staff enables me to pay more attention to the clinical care.'* (CD 2)

The provision of infantometers and weighing machines for active child growth monitoring in the trial created a ripple effect at these clinics, as other mothers (not registered in the trial) also started requesting for their children to be measured; this indicated a general interest of mothers to know more about their child’s wellbeing:

*'Mothers bringing child to the clinic generally asks how their child is growing.*' (CD 4)

Recording of child development milestones was not a requirement of ECD care delivery in the trial. Therefore, it was not possible to assess the providers’ adherence to care protocols and child referrals.

#### Other

Most clinics waivered their consultation fee for the ECD care follow-up visits, on request of the district coordinator from respective DHOs. This was a good-intention gesture, but seemed to have also been a considered decision to build rapport and promote business-satisfied clients referring their friends to the clinic:

*'Offering subsidised care is a recognised approach to build clinic rapport.'* (CD 2)

The continued engagement of the DHO, especially during trouble-shooting clinics deviating from care protocols, was acceptable and feasible for both DHOs and private clinics. Although the performance level (case registration and follow-up) varied across clinics, none of the initially included clinics left the intervention.

### Continuity of care

#### Follow-up by private clinic

The review of 1242 child intervention records showed that about one-third (34%) of mother–child pairs made three or more follow-up visits (maximum recommended visit: four), more than half (54%) came for one or two follow-up visits, and about one-tenth (12%) did not attend any follow-up visit. The clinic staff calling to remind the due follow-up visit, on a mutually agreed phone number, was found feasible for the clinic staff and acceptable to the families:

*'No problem in contacting a mother on her family mobile, preferably when her husband is at home.'* (CA 2)

The clinic staff reported only occasional difficulty in making contact on the given phone numbers for example, found not in operation. The main reasons for mothers’ non-adherence to the ECD care follow-up visits were lack of perceived need, such as the child is doing fine and/or family knows what to do for child care, and mothers’ difficulty in managing time for the follow-up visits, for example clinic timing not convenient for her to attend. Geographical and financial accessibility were not mentioned as challenges for mothers to adhere to the follow-up visit schedule for ECD care:

*'My mother in-law believes that her on-hand experience of decades is enough for taking good care of our child.'* (Mother 5, Clinic 1)

#### Referral by private clinic

The experience of establishing a referral link between the private clinics and public specialist institutions faced multiple challenges including:

- institutional procedural requirements and lack of desired flexibility to entertain referrals differently (as per clinic and or client expectation);
- clinic ignorance and/or reluctance to refer clients for specialised care;
- client reluctance (due to social acceptance and financial apprehension); and
- client inability (due to geographic and resource constraints) to attend the specialised ECD care at a distant institution.

*'Some of my clients show reluctance to get specialist care from a public institution because they feel uncertain about the access and cost of care.'* (CD 1)

## Discussion

### Summary

The process evaluation allowed insight in to the mechanisms for public–private partnership for delivering ECD care at GP private clinics. The intervention components were feasible for both providers (private clinics) and clients (mothers). The existing clinic set up was feasible (with minimal input of child measurement equipment) for conducting an ECD counselling session; clinic assistants found the pictorial counselling tool a helpful aid in delivering comprehensible care.

DHO involvement proved to be beneficial for:

- transparent and effective appointment selection of private clinics;
- training facilitated by public specialists in ECD care;
- endorsement to promote uptake of ECD care uptake by mothers; and 
- engagement of community advocates for promotion of ECD uptake at specified clinics.

The continued engagement of the DHO not only minimised deviation from care protocols but also encouraged clinics to waive follow-up fees for mothers seeking ECD counselling. However, adherence to care protocols for child development screening at follow-up visit could not be accounted for due to less emphasis on recording keeping on child development achievement. Mothers were consistent in returning for their follow-up visits; moreover, referral for specialist consultation could not be streamlined due to challenges posed by provider and client perceptions and expectations along with difficulty in setting up a pathway in the respective referral institutions.

### Strengths and limitations

The approach to learn about both the provider and the consumer experiences of each care task has been helpful to assess if the operational strategies for ECD care delivery were kept sensitive to the provider and the consumer circumstances and preferences.

However, the key study limitations include the available clinical record limited the choice of quantitative indicators, a sample of retrievable mothers was interviewed, so experiences of mothers that could not be retrieved are not represented, mother interviews focused more on their access and use of clinic-based ECD care, with limited data on their household circumstances and practices related to ECD care.

This early experience indicates that with a minimal programme investment, to train staff and provide minor equipment, the private clinics can be effectively engaged in delivering clinic-based ECD care in poor urban settings; where coverage of public health care is known to be scant.

### Comparison with existing literature and implications

Public–private partnerships have proven to be effective in improving quality and provision of maternal child services in various low- and middle-income countries, including Guatemala, Bolivia and Haiti.^15–17^ A combination of objective information (clinic survey) and experience-wisdom (district officials) helped identifying suitable clinics for inclusion in a public–private partnership intervention. In the partnership (rather than regulation) approach, it is important for the district health office and the identified clinics to take informed decisions about their participation in an intervention. This approach to transparent selection and recruitment of private clinics is also being implemented for scaling the communicable disease (such as tuberculosis and malaria) interventions in Pakistan.^7–8,18^ The public endorsement of private clinics is an important consideration; the provincial government has formed an independent body, Punjab Health Care Commission, to assess and certify public and private healthcare facilities for delivering various healthcare packages. In the future, offering programme-endorsed care would become more important for the clinics to qualify their certification.^19^

The private clinics were interested and able to deliver health promotion care within their respective settings. The compatibility of ECD care with their settings and business model indicates the potential of expanding the scope of health promotion activities at private clinics. The public support seems important for the private providers to get updated about best prevention and treatment practices.^20^ Public sector training of private healthcare providers has been successfully modelled in other countries of the region including India, Nepal, and Bangladesh.^21–24^

The engagement of community advocates was useful in promoting the uptake of ECD care at the clinics, as found in other community-based interventions.^25–27^ With about one-quarter of registration attributed to community advocates’ referrals, the clinic regular clientele seemed to have played much important role in the uptake of ECD care at the respective clinic. Thus, prioritising the clinics with higher number of existing mother and child clients can potentially increase the efficiency of programme/district investment in public–private partnership for ECD care.

Mothers’ acceptance of clinic-based ECD care indicates that an intervention kept sensitive to the cultural preferences can help overcoming possible cultural barriers implied in similar health programmes.^28–30^ A take-home brochure, with the key recommended actions included, seems to have supplemented the clinic-based counselling. It also helped ECD care at home, regardless of mother being adherent or less adherent to the quarterly clinic visits. Experiences in other projects also report similar usefulness of a pictorial tool to promote home-based care practices.^31–34^

Establishing an effective and potentially replicable referral arrangement for specialist ECD care was found rather challenging. Consistent with research, the complex undertaking of establishing a referral linkage between private clinic and public institution needs more concerted efforts to understand and address the political, social, administrative, and financial challenges.^35^

## Trial registration

The trial has been registered with the Current Controlled Trials ISRCTN48032200.

## References

[1] Grantham-McGregor S, Cheung YB, Cueto S (2007). Developmental potential in the first 5 years for children in developing countries. The Lancet.

[2] Black MM, Walker SP, Wachs TD (2008). Policies to reduce undernutrition include child development. The Lancet.

[3] WHO Department of Child and Adolescent Health and Development (1999.). A critical link: interventions for physical growth and psychological development: a review.

[4] Hirani SA (2012). Malnutrition in young Pakistani children. JAMC.

[5] Murray L, Cooper P (1997). Effects of postnatal depression on infant development. Arch Dis Child.

[6] Patel V, Rahman A, Jacob KS (2004). Effect of maternal mental health on infant growth in low income countries: new evidence from South Asia. BMJ.

[7] National TB control programme Pakistan, Technical Assistance Management Agency (2017). Situation analysis, public–private partnership models, operational and monitoring and evaluation guidelines for national TB control programme Pakistan. http://ntp.gov.pk/uploads/ntp_1369819747_Finalreport_ppp_NTP08_09_06.pdf.

[8] Directorate of Malaria Control, Pakistan. Ministry of National Health Services, Regulation & Coordination. (2017). Pakistan Malaria Programme Review (MPR). http://dmc.gov.pk/documents/pdfs/4MPR%20Pakistan.pdf.

[9] Khan MA, Javed W, Ahmed M (2013). Delivering enhanced cardiovascular (hypertension) disease care through private health facilities in Pakistan. BMC Cardiovasc Disord.

[10] Khan MA, Owais SS, Blacklock C (2017). Delivering integrated child development care in Pakistan: protocol for a clustered randomised trial. BJGP Open.

[11] Moore GF, Audrey S, Barker M (2015). Process evaluation of complex interventions: Medical Research Council guidance. BMJ.

[12] Creswell JW, Klassen AC, Plano VL (2011). Best practices for mixed methods research in the health sciences.

[13] Craig P, Dieppe P, Macintyre S (2008). Developing and evaluating complex interventions: the new Medical Research Council guidance. BMJ.

[14] Gale NK, Heath G, Cameron E (2013). Using the framework method for the analysis of qualitative data in multi-disciplinary health research. BMC Med Res Methodol.

[15] Danel I, La Forgia FM, La Forgia GM (2005).

[16] Lavadenz F, Schwab N, Straatman H (2001). [Public, decentralized and community health networks in Bolivia]. Rev Panam Salud Publica.

[17] Eichler R, Auxila P, Pollack J (2017). Performance-based payment to improve the impact of health services: evidence from Haiti.. http://www1.paho.org/hq/dmdocuments/2010/44-Performance_Based_Reimbursement_Improve_Impact-Haiti.pdf.

[18] Baloch NA, Pai M (2012). Tuberculosis control: business models for the private sector. Lancet Infect Dis.

[19] Punjab Healthcare Commission (2013). Clinicalgovernance.

[20] Smith E, Brugha R, Zwi A (2017). Working with private sector providers for better health care: an introductory guide. London School of Hygiene and Tropical Medicine. http://www.who.int/management/partnerships/private/privatesectorguide.pdf.

[21] Khan MS, Salve S, Porter JD (2015). Engaging for-profit providers in TB control: lessons learnt from initiatives in South Asia. Health Policy Plan.

[22] USAID (2015). Private Sector Mobilization for family health — Phase 2 (PRISM2) Project. http://pdf.usaid.gov/pdf_docs/PA00K9HD.pdf.

[23] Mash R, Almeida M, Wong WC (2015). The roles and training of primary care doctors: China, India, Brazil and South Africa. Human Resources for Health.

[24] Newell JN, Pande SB, Baral SC (2004). Control of tuberculosis in an urban setting in Nepal: public–private partnership. Bulletin of the World Health Organization.

[25] UNICEF (2012). Early childhood — advocacy and partnerships for early childhood development. https://www.unicef.org/earlychildhood/index_40753.html.

[26] McPherson RA, Tamang J, Hodgins S (2010). Process evaluation of a community-based intervention promoting multiple maternal and neonatal care practices in rural Nepal. BMC Pregnancy and Childbirth.

[27] Farnsworth SK, Böse K, Fajobi O (2014). Community engagement to enhance child survival and early development in low- and middle-income countries: an evidence review. J Health Commun.

[28] Winch PJ, Alam MA, Akther A (2005). Local understandings of vulnerability and protection during the neonatal period in Sylhet District, Bangladesh: a qualitative study. The Lancet.

[29] Nichter M (1987). Cultural dimensions of hot, cold and sema in Sinhalese health culture. Soc Sci Med.

[30] Darmstadt GL, Saha SK (2002). Traditional practice of oil massage of neonates in Bangladesh. J Health, Popul Nutr.

[31] WHO, UNICEF (2017). http://www.who.int/mental_health/emergencies/ecd_note.pdf .

[32] Edward-Raj A, Phiri R (2017). http://pdf.usaid.gov/pdf_docs/Pnacs120.pdf.

[33] Leshabari S, Koniz-Booher P, Burkhalter B (2007). . Testing a PMTCT infant-feeding counseling program in Tanzania. Operations research results.. https://www.usaidassist.org/sites/assist/files/orrtztestingjobaids.pdf .

[34] Zaman S, Ashraf RN, Martines J (2008). Training in complementary feeding counselling of healthcare workers and its influence on maternal behaviours and child growth: a cluster-randomized controlled trial in Lahore, Pakistan. Journal of Health, Population, and Nutrition.

[35] Ministry of Health, Kenya. (2013. ). https://www.measureevaluation.org/pima/baseline-assessments/the-state-of-the-health-referral-system-in-kenya-results-from-a-baseline-study-on-the-functionality-of-the-health-referral-system-in-eight-counties/view.

